# Tests of fit for the power function lognormal distribution

**DOI:** 10.1371/journal.pone.0298309

**Published:** 2024-02-22

**Authors:** Chao Wang, He Zhu

**Affiliations:** 1 School of Mathematics and Statistics, Anyang Normal University, Anyang, China; 2 School of Statistics and Mathematics, Zhejiang Gongshang University, Hangzhou, China; Abdul Wali Khan University Mardan, PAKISTAN

## Abstract

In this study, tests of fit for the power function lognormal distribution is considered. The probability plot, probability plot correlation coefficient, and goodness-of-fit tests—the Kolmogorov–Smirnov (KS), Cramér–von Mises (CvM), and Anderson–Darling (AD) tests are provided. Tables of critical values are presented by using simulation techniques, and the AD test outperforms KS and CvM tests based on power comparisons. Finally, to illustrate these test procedures, we fit this distribution to the data which represent the survival times of 121 breast cancer patients from one hospital.

## 1. Introduction

Statistical distributions are important for modeling and predicting real-world situations. For example, the empirical analysis of the distribution of income amounts is a major topic of research in development economics. One of the primary purposes of this analysis is simply to describe the distribution of income and derive descriptive and summative inequality measures, such as the Gini coefficient. A large number of income distributions have been proposed in the statistical literature, including the lognormal, gamma, Pareto, Weibull, Dagum, Singh–Maddala, and generalized beta–2 distributions [1].

Another type of income distribution, the power function lognormal composite (PFLC) distribution, has recently been proposed [2]. This flexible distribution can have positive or negative skewness and can be either leptokurtic or platykurtic, depending on the parameters. Extensive statistical inference methods have also been presented [2], such as those for modeling household income and automobile insurance claims. Moreover, it has been demonstrated that inequality measures, including the Gini coefficient, generalized entropy index, Theil’s entropy index, and the Atkinson, Bonferroni, and Zenga indexes, can be obtained using numerical methods based on the PFLC distribution [3].

The modeling and analysis of lifetime data are important aspects of statistical work in areas such as engineering, medicine, and the biological sciences. Lifetime models are often skewed or are far from normal. Therefore, models such as the exponential, Weibull, lognormal, and gamma often occupy a central position because of their demonstrated usefulness in a wide range of situations. Over the years, many models have been developed and applied to lifetime data, including the exponentiated generalized linear exponential distribution [4], generalized transmuted-G family [5], modified beta transmuted exponential distribution [6], extended gumbel distribution [7], and generalized Marshall-Olkin exponentiated exponential distribution [8]. We will demonstrate that the PFLC distribution can also be applied to the modeling of lifetime data.

Goodness-of-fit (GoF) usually refers to whether a dataset is consistent with sampling from a model for a distribution. Many GoF tests exist, and they can generally be classified as either graphical techniques or statistical methods. Statistical methods are usually preferred because of their objectivity. One frequently used graphical method is the probability plot [9], which generally uses special scales on which the cumulative distribution function (CDF) of a particular distribution plots as a straight line. A normal probability plot is defined as a plot of the *i*th-order statistic versus some measure of location of the *i*th-order statistics from a standard normal distribution. The probability plot correlation coefficient, the product moment correlation coefficient that measures the degree of linear association between these two random variables, can be used as an appropriate test statistic [10]. The Kolmogorov–Smirnov (KS), Cramér–von Mises (CvM), and Anderson–Darling (AD) tests are but a few of the traditional statistical tests available to determine GoF. These are all based on the empirical distribution function (EDF). They test the null hypothesis by measuring the distance between the EDF estimated from observed data and the CDF of the fitted models [11].

We introduce and implement probability plot, probability plot correlation coefficient, and GoF tests for the PFLC distribution. The first two methods are based on a transformation of the cumulative distributions. The classical KS, CvM, and AD tests are considered. A power study was conducted to investigate their performance, and the results show that the AD test outperforms the KS and CvM tests, and that the KS test is the weakest, requiring a much larger sample size to achieve comparable power to the other tests. We also show that the PFLC distribution can provide a good fit for one survival dataset.

The rest of this paper is organized as follows. The setup and statistical properties of the PFLC distribution are discussed in Section 2. The probability plot method and probability plot correlation coefficient are investigated in Sections 3 and 4, respectively. The derivations of computing formulae, critical values, and power comparisons for three PFLC GoF test statistics follow in Section 5. An application to one survival time is presented in Section 6. Some conclusions are drawn in Section 7.

## 2. Power function lognormal composite distribution

The probability density function (PDF) of the PFLC distribution can be written as [2]

$$f\left(x\right)=\left\{\begin{array}{ll} w\alpha x^{\alpha -1}/\theta^{\alpha }, & 0<x\leq \theta \\ \frac{\left(1-\text{w}\right)}{\Phi \left(\alpha \sigma \right)\sqrt{2\pi }x\sigma }exp\left[-\frac{1}{2}{\left(\frac{lnx-ln\theta -\alpha \sigma^{2}}{\sigma }\right)}^{2}\right], & \theta \leq x<+\infty \end{array},\right.$$ (1)

where *w* is a mixing weight, defined as

$$w=\frac{1}{1+\sqrt{2\pi }a\sigma \Phi \left(\alpha \sigma \right)exp\left[0.5{\left(\alpha \sigma \right)}^{2}\right]},$$ (2)

and Φ(.) is the CDF of the standard normal distribution.

PFLC is a distribution in three unknown parameters—α > 0, θ > 0, and σ > 0—and X~PFLC(*α*,*θ*,*σ*) indicates that *X* follows this distribution.

The corresponding CDF *F*(*x*), and the quantile function *X*(*p*) are given by

$$f\left(x\right)=\left\{\begin{array}{ll} w{\left(x/\theta \right)}^{\alpha }, & 0<x\leq \theta \\ 1-\frac{\left(1-\text{w}\right)}{\Phi \left(\alpha \sigma \right)}\left[1-\Phi \left(\frac{lnx-ln\theta -\alpha \sigma^{2}}{\sigma }\right)\right], & \theta \leq x<+\infty \end{array},\right.$$ (3)

and

$$X\left(p\right)=\left\{\begin{array}{ll} \theta {\left(p/w\right)}^{1/\alpha }, & 0<p\leq w\\ \theta exp\left\{\sigma \Phi^{-1}\left[1-\left(1-p\right)\Phi \left(\alpha \sigma \right)/\left(1-w\right)\right]+\alpha \sigma^{2}\right\}, & w\leq p<1 \end{array}.\right.$$ (4)

Let X~PFLC(α,θ,σ). Then *Y* = *w*(*X*/*θ*)^*α*^ has a density function

$$f\left(y\right)=\left\{\begin{array}{ll} 1, & 0<y\leq w\\ \frac{\left(1-w\right)}{\Phi \left(\alpha \sigma \right)\sqrt{2\pi }\left(\alpha \sigma \right)y}exp\left[-\frac{1}{2}\frac{{\left(lny-lnw-\alpha^{2}\sigma^{2}\right)}^{2}}{{\left(\alpha \sigma \right)}^{2}}\right], & w\leq y<+\infty \end{array}.\right.$$ (5)

From (2), one can easily verify that *w* is a decreasing function of *ασ*. Table 1 presents the values of *ασ* for a grid of *w* values [0.01(0.01)0.99], which are accurate to about six significant digits. Thus *f*(*y*) in (5) can also be seen as a function of *y* when *w* is given.

**Table 1:** Values of *ασ* for a grid of *w* values.

*w*	*ασ*	*w*	*ασ*	*w*	*ασ*	*w*	*ασ*	*w*	*ασ*
**0.01**	2.374888	**0.21**	1.034510	**0.41**	0.635877	**0.61**	0.369761	**0.81**	0.163452
**0.02**	2.116740	**0.22**	1.008522	**0.42**	0.620425	**0.62**	0.358324	**0.82**	0.154183
**0.03**	1.955544	**0.23**	0.983467	**0.43**	0.605255	**0.63**	0.347024	**0.83**	0.144997
**0.04**	1.835927	**0.24**	0.959271	**0.44**	0.590355	**0.64**	0.335859	**0.84**	0.135892
**0.05**	1.739836	**0.25**	0.935869	**0.45**	0.575715	**0.65**	0.324824	**0.85**	0.126867
**0.06**	1.659007	**0.26**	0.913203	**0.46**	0.561323	**0.66**	0.313916	**0.86**	0.117919
**0.07**	1.588934	**0.27**	0.891222	**0.47**	0.547170	**0.67**	0.303132	**0.87**	0.109048
**0.08**	1.526878	**0.28**	0.869879	**0.48**	0.533247	**0.68**	0.292467	**0.88**	0.100251
**0.09**	1.471043	**0.29**	0.849132	**0.49**	0.519544	**0.69**	0.281919	**0.89**	0.091528
**0.10**	1.420187	**0.30**	0.828945	**0.50**	0.506054	**0.70**	0.271485	**0.90**	0.082876
**0.11**	1.373412	**0.31**	0.809282	**0.51**	0.492770	**0.71**	0.261162	**0.91**	0.074294
**0.12**	1.330048	**0.32**	0.790114	**0.52**	0.479682	**0.72**	0.250947	**0.92**	0.065781
**0.13**	1.289580	**0.33**	0.771412	**0.53**	0.466786	**0.73**	0.240837	**0.93**	0.057336
**0.14**	1.251604	**0.34**	0.753150	**0.54**	0.454074	**0.74**	0.230829	**0.94**	0.048956
**0.15**	1.215797	**0.35**	0.735304	**0.55**	0.441539	**0.75**	0.220922	**0.95**	0.040642
**0.16**	1.181896	**0.36**	0.717854	**0.56**	0.429177	**0.76**	0.211113	**0.96**	0.032391
**0.17**	1.149685	**0.37**	0.700778	**0.57**	0.416981	**0.77**	0.201399	**0.97**	0.024202
**0.18**	1.118983	**0.38**	0.684059	**0.58**	0.404947	**0.78**	0.191778	**0.98**	0.016075
**0.19**	1.089637	**0.39**	0.667680	**0.59**	0.393069	**0.79**	0.182248	**0.99**	0.008008
**0.20**	1.061516	**0.40**	0.651624	**0.60**	0.381342	**0.80**	0.172807		

The CDF *F*(*y*) and quantile function *Y*(*p*) are given by

$$F\left(y\right)=\left\{\begin{array}{ll} y, & 0<y\leq w\\ 1-\frac{\left(1-\text{w}\right)}{\Phi \left(\alpha \sigma \right)}\left[1-\Phi \left(\frac{\left(lny-lnw-\alpha^{2}\sigma^{2}\right)}{\alpha \sigma }\right)\right], & w\leq y<+\infty \end{array},\right.$$ (6)

and

$$Y\left(p\right)=\left\{\begin{array}{ll} p, & 0<p\leq w\\ exp\left\{lnw+\alpha^{2}\sigma^{2}+\alpha \sigma \Phi^{-1}\left[1-\frac{\left(1-p\right)}{\left(1-w\right)}\Phi \left(\alpha \sigma \right)\right]\right\}, & w\leq p<1 \end{array}.\right.$$ (7)

We will refer to distribution (5) as the standard power function lognormal composite distribution, denoted by SPFLC(w).

## 3. Probability plot

Taking the logarithm of (4), we can obtain

$$lnX=\left\{\begin{array}{ll} ln\theta +1/\alpha ln\left(p/w\right), & 0<p\leq w\\ ln\theta +\sigma \Phi^{-1}\left[1-\left(1-p\right)\Phi \left(\alpha \sigma \right)/\left(1-w\right)\right]+\alpha \sigma^{2}, & w\leq p<1 \end{array}.\right.$$ (8)

Note that when *p* = *w*, *lnθ* + 1/*α ln*(*p*/*w*) = *lnθ* + 1/*α ln*(*p*/*p*) = *lnθ*, and *lnθ* + *σ*Φ^−1^ [1 − (1 − *p*) Φ(*ασ*)/(1 − *w*)] + *ασ*^2^ = *lnθ* + *σ*Φ^−1^ [Φ(−*ασ*)] + *ασ*^2^ = *lnθ*.

Eq (8) can be written as

$$\left\{\begin{array}{ll} \frac{1}{\alpha }\left[lnp-lnw\right]=-ln\theta +lnX, & 0<x\leq \theta \\ \sigma \Phi^{-1}\left[1-\left(1-p\right)\Phi \left(\alpha \sigma \right)/\left(1-w\right)\right]+\alpha \sigma^{2}=-ln\theta +lnX, & \theta \leq x<+\infty \end{array}.\right.$$ (9)

*q* can be considered to be equal to the following formula:

$$q=\left\{\begin{array}{ll} \frac{1}{\alpha }\left[lnp-lnw\right], & 0<p\leq w\\ \sigma \Phi^{-1}\left[1-\left(1-p\right)\Phi \left(\alpha \sigma \right)/\left(1-w\right)\right]+\alpha \sigma^{2}, & w\leq p<1 \end{array}.\right.$$ (10)

Thus, (9) represents a linear relationship between *q* and *lnx*, with an intercept of −*lnθ* and a slope of 1.

**Fig 1:**
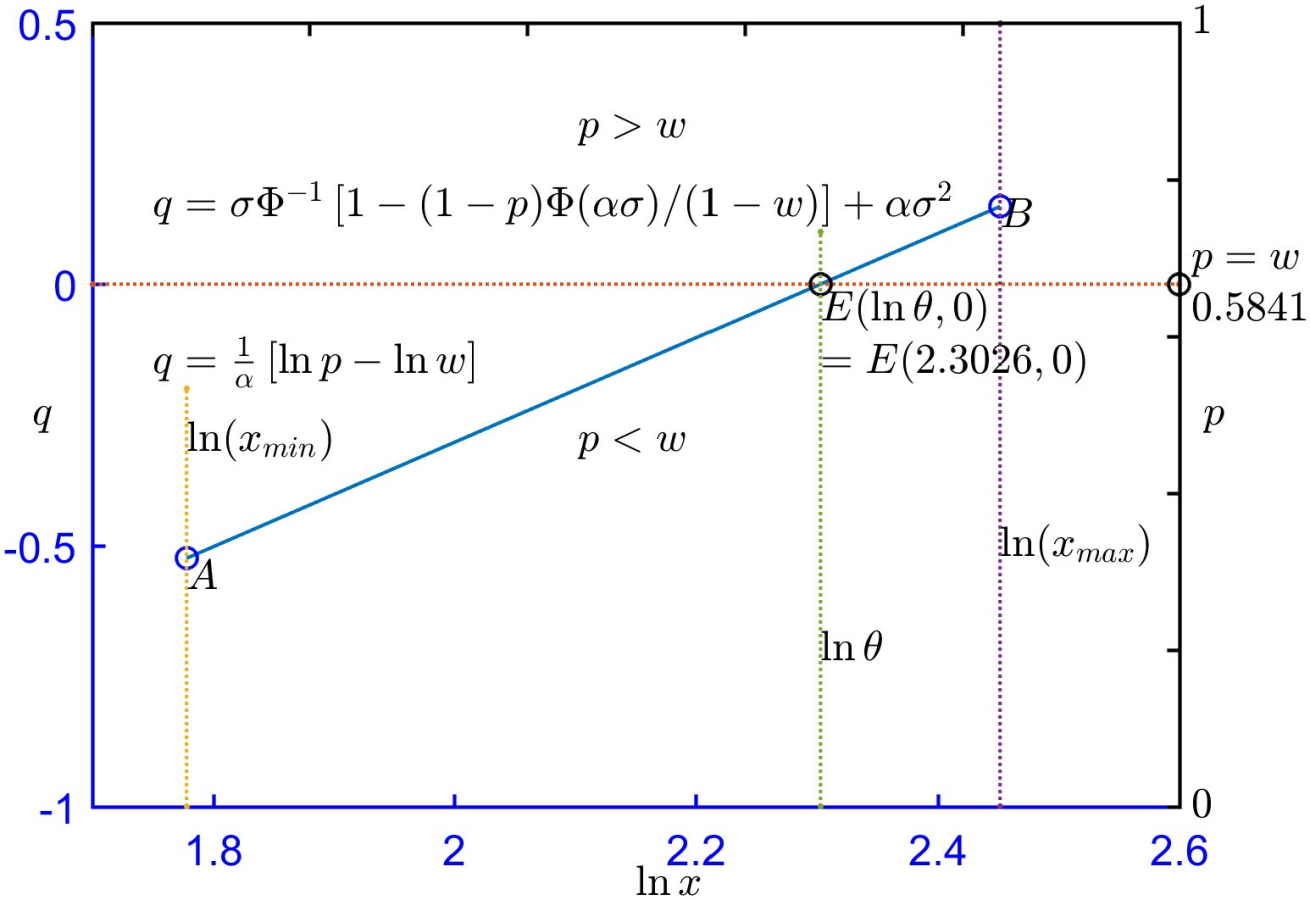
Probability plot for 100 sets of simulated PFLC(5,10,0.08) data.

The probability plot of 100 sets of simulated data from PFLC(5,10,0.08) with random seed 1234 is shown in Fig 1, where the solid line *AB* is a probability plot drawn according to (8). The starting point $A\left(\text{ln}\left(x_{min}\right),\text{ln}\left(\frac{x_{min}}{\theta }\right)\right)$ is the intersection of *lnx* = ln(*x*_*min*_) and *AB*, where ln(*x*_*min*_) is the natural logarithm of the minimum value of the simulated data. The ending point $B\left(\text{ln}\left(x_{max}\right),\text{ln}\left(\frac{x_{max}}{\theta }\right)\right)$ is the intersection of *lnx* = ln(*x*_*max*_) and *AB*, where ln(*x*_*max*_) is the natural logarithm of the maximum value of the simulated data. Point *E*(*lnθ*, 0) is the intersection of *lnx* = *lnθ* and *q* = 0, which corresponds exactly to *p* = *w*. In this example, ln(*x*_*min*_) = 1.7780, ln(*x*_*max*_) = 2.4514, and $\text{w}={\left\{1+\sqrt{2\pi }\alpha \sigma \Phi \left(\alpha \sigma \right)exp\left[0.5{\left(\alpha \sigma \right)}^{2}\right]\right\}}^{-1}={\left\{1+\sqrt{2\pi }0.4\Phi \left(0.4\right)exp\left[0.5{\left(0.4\right)}^{2}\right]\right\}}^{-1}=0.5841$.

The line *q* = 0 divides *AB* into two parts. For the upper part *BE*, *q* = *σ*Φ^−1^ [1 − (1 − *p*) Φ(*ασ*)/(1 − *w*)] + *ασ*^2^, and for the lower part *AE*, *q* = 1/*α* [*lnp* − *lnw*]. The straight line *p* = 0.5841 also divides *AB* into two parts: *p* < 0.5841 and *p* > 0.5841.

Many authors have discussed methods for choosing the values of *p* for a given *n* for use in such plots [9]. We use the Hazen formula,

$$p_{i}=\frac{i-0.5}{n},i=1,2,\cdots ,n.$$ (11)

We determine whether the PFLC distribution can be used to fit one dataset using a probability plot as follows:

(i) Order the sample values to obtain *x*_1_ ≤ *x*_2_ ≤ ⋯ ≤ *x*_*n*_.
(ii) Obtain the estimated parameters $\left(\hat{\alpha },\hat{\theta },\hat{\sigma }\right)$ for (*α*, *θ*, *σ*) by maximum likelihood, and then compute $\hat{w}={\left\{1+\sqrt{2\pi }\hat{\alpha }\hat{\sigma }\Phi \left(\hat{\alpha }\hat{\sigma }\right)exp\left[0.5{\left(\hat{\alpha }\hat{\sigma }\right)}^{2}\right]\right\}}^{-1}$.
(iii) Compute *q* from (10), where $\;p_{i}=\frac{i-0.5}{n},i=1,2,\cdots ,n$.
(iv) Plot *q* versus *lnx*; the PFLC distribution can be used to model the dataset if the plot is approximately a straight line.

**Fig 2:**
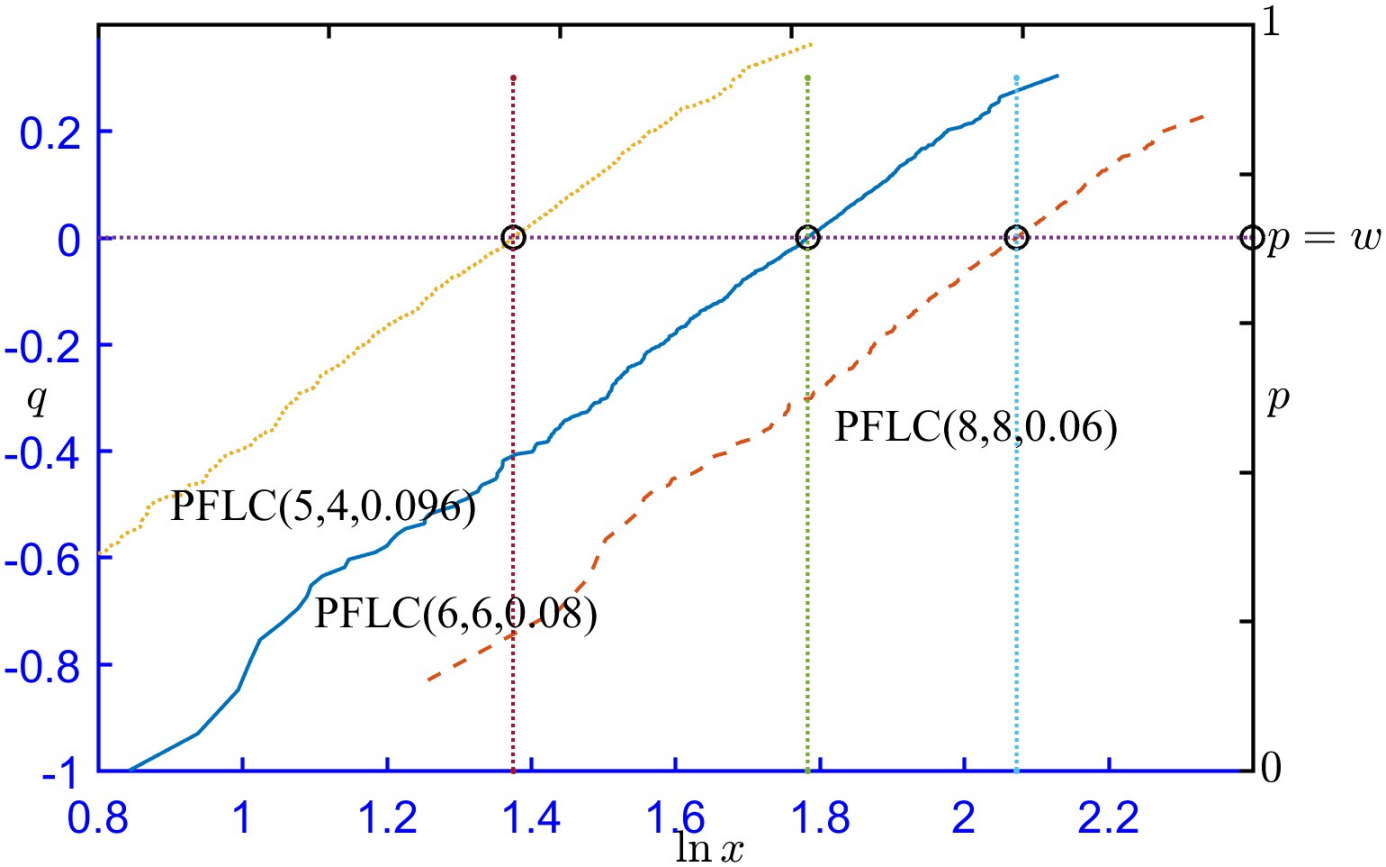
Probability plots for three simulated PFLC datasets.

Fig 2 illustrates the probability plots of simulated data from PFLC(5,4,0.096), PFLC(6,6,0.08), and PFLC(8,8,0.06). For each case, 1000 sets of data are generated with random seed 1234. Fig 2 shows that the plots are approximately linear, which indicates that the underlying distributions are PFLCs.

## 4. The probability plot correlation coefficient

The probability plot correlation (PPC) coefficient was used as a test statistic for normality [10]. The PPC test measures the linearity of a probability plot; if the probability plot is expected to be almost linear, then the correlation coefficient will be close to one. We then derive the PPC test for the PFLC distribution.

Taking the logarithm of (7), we obtain

$$lnY\left(p\right)=\left\{\begin{array}{ll} lnp, & 0<p\leq w\\ lnw+\alpha^{2}\sigma^{2}+\alpha \sigma \Phi^{-1}\left[1-\frac{\left(1-p\right)}{\left(1-w\right)}\Phi \left(\alpha \sigma \right)\right], & w\leq p<1 \end{array}.\right.$$ (12)

Letting *Z*_*i*_ = *lnY*_*i*_, then *T*_1*i*_, *T*_2*i*_ are defined as

$$\left\{\begin{array}{ll} T_{1i}=ln\left[\left(i-0.5\right)/n\right], & 0<i\leq m\\ T_{2i}=lnw+\alpha^{2}\sigma^{2}+\alpha \sigma \Phi^{-1}\left[1-\frac{\left(1-\left(i-0.5\right)/n\right)}{\left(1-w\right)}\Phi \left(\alpha \sigma \right)\right], & m+1\leq i\leq n \end{array}.\right.$$ (13)

Then, the correlation coefficient *r*_*Q*_ between *Z*_*i*_ and *T*_*i*_ can be defined as

$$r_{Q}=\frac{\sum_{i=1}^{m}\left(T_{1i}-\bar{T}\right)\left(Z_{i}-\bar{Z}\right)+\sum_{i=m+1}^{m}\left(T_{2i}-\bar{T}\right)\left(Z_{i}-\bar{Z}\right)}{\sqrt{\sum_{i=1}^{m}{\left(T_{1i}-\bar{T}\right)}^{2}+\sum_{i=m+1}^{n}{\left(T_{2i}-\bar{T}\right)}^{2}}\sqrt{\sum_{i=1}^{m}{\left(Z_{i}-\bar{Z}\right)}^{2}}}.$$ (14)

Because *r*_*Q*_ only depends on *w*, we can obtain the critical values of this statistic for a given *w* in practice. Monte Carlo studies have determined the percentage points of the statistics for sample sizes *n* = 5(5)90, 90(10)100, 100(50)500, and 500(100)1000. For each case, the procedure was repeated 20 000 times to produce an empirical distribution of the test statistic, from which sample quantiles approximating the critical values were obtained. For *w* = 0.1, the algorithm to obtain the 5% critical values is as follows.

(i) Set *w* = 0.1, generate *n* random numbers from (7), and order the sample values to obtain *y*_1_ ≤ *y*_2_ ≤ ⋯ ≤ *y*_n_. Then, obtain ln(*y*_1_) ≤ ln(*y*_2_) ≤ ⋯ ≤ ln(*y*_*n*_).
(ii) Compute *m*, which is the number of *y*_*i*_ values less than *w*, and compute percentile point *p*_*i*_ from (11).
(iii) Compute *αθ* from (2), and then compute *T*_1*i*_ and *T*_2*i*_ from (13).
(iv) Compute the correlation coefficient *r*_*Q*_ between *Z*_*i*_ and *T*_*i*_ from (14).
(v) Repeat steps (i)-(iv) 20000 times to obtain the 5% sample quantiles of *r*_*Q*_ as the 5% critical values.

Table 2 presents the 5% critical values of the distribution of *r*_*Q*_ for selected sample sizes when *w* equals 0.1(0.2)0.9. For example, the critical value of *r*_*Q*_ for *n* = 10 is 0.910701 when *w* is 0.1; this means that in 10% of random samples of size 10, the correlation coefficient will be at least 0.910701.

**Table 2:** 5% critical values of *r*_*Q*_ under different *w* values.

*n*	0.1	0.3	0.5	0.7	0.9
**5**	0.878900	0.881580	0.858793	0.836375	0.843420
**10**	0.910701	0.904921	0.899432	0.895491	0.894638
**15**	0.927814	0.922509	0.916011	0.915398	0.916078
**20**	0.928705	0.931601	0.927854	0.929216	0.926918
**25**	0.950457	0.941754	0.937833	0.936049	0.936141
**30**	0.956187	0.947413	0.943733	0.943238	0.941715
**35**	0.960655	0.950716	0.948029	0.947828	0.945738
**40**	0.962947	0.953929	0.952719	0.951541	0.950341
**45**	0.965731	0.958320	0.954487	0.954225	0.954616
**50**	0.968940	0.961557	0.957077	0.957447	0.956504
**55**	0.970011	0.962392	0.961351	0.959968	0.959402
**60**	0.971477	0.966481	0.962725	0.961047	0.961045
**65**	0.973303	0.966768	0.964068	0.962321	0.963230
**70**	0.975489	0.968009	0.965907	0.965082	0.963203
**75**	0.976720	0.969635	0.967489	0.966385	0.966691
**80**	0.977327	0.971138	0.968883	0.967268	0.966725
**85**	0.978128	0.972253	0.969186	0.969221	0.969280
**90**	0.978508	0.973148	0.970487	0.970542	0.970154
**100**	0.980343	0.975576	0.972893	0.972090	0.972450
**150**	0.986075	0.981590	0.979519	0.978228	0.977921
**200**	0.988897	0.984717	0.983278	0.982538	0.981999
**250**	0.990474	0.987825	0.985843	0.984351	0.984806
**300**	0.991784	0.988749	0.987397	0.986933	0.986218
**350**	0.992947	0.989920	0.989146	0.988097	0.988215
**400**	0.993538	0.991077	0.989663	0.989575	0.989296
**450**	0.994181	0.991836	0.990808	0.990080	0.990309
**500**	0.994630	0.992453	0.991640	0.990895	0.990710
**600**	0.995397	0.993524	0.992541	0.992013	0.991897
**700**	0.995935	0.994036	0.993303	0.993074	0.992799
**800**	0.996300	0.994848	0.994107	0.993652	0.993494
**900**	0.996683	0.995433	0.994473	0.994498	0.994262
**1000**	0.997053	0.995594	0.995106	0.994625	0.994647

We can determine whether the PFLC distribution can be used to fit one dataset by the correlation coefficient at 5% significance level as follows:

(i) Order the sample values to obtain *x*_1_ ≤ *x*_2_ ≤ ⋯ ≤ *x*_n_.
(ii) Obtain the estimated parameters $\left(\hat{\alpha },\hat{\theta },\hat{\sigma }\right)$ for (*α*, *θ*, *σ*) by maximum likelihood, and calculate $\hat{w}={\left\{1+\sqrt{2\pi }\hat{\alpha }\hat{\sigma }\Phi \left(\hat{\alpha }\hat{\sigma }\right)exp\left[0.5{\left(\hat{\alpha }\hat{\sigma }\right)}^{2}\right]\right\}}^{-1}$.
(iii) Calculate *Z*_*i*_ = *lny*_*i*_, where $y_{i}=\hat{w}{\left(x_{i}/\hat{\theta }\right)}^{\hat{\alpha }},i=1,2,\cdots ,n$.
(iv) Calculate *r*_*Q*_ from (14).
(v) Reject *H*_0_ (the sample is from a PFLC distribution) at 5% significance level if *r*_*Q*_ is less than the 5% critical values.

## 5. Goodness-of-fit tests

We discuss three GoF for the PFLC distribution [11]. As described in Section 2, the test for X~PFLC(α,θ,σ) is equivalent to that for Y~SPFLC(*w*). Therefore we test whether the underlying probability distribution is SPFLC(*w*) for a given random sample *Y*_1_, *Y*_2_, ⋯, *Y*_*n*_.

### 5.1. Basic GoF test statistics

Let *y*_1_, *y*_2_, ⋯, *y*_*n*_ denote the set of the original data in ascending order. The test statistic for the KS test is thus

$$D=\text{max}\left(D^{-},D^{+}\right),$$ (15)

where

$$D^{-}=\underset{1\leq i\leq n}{\text{max}}\left\{\hat{F}\left(y_{i}\right)-\frac{i-1}{n}\right\}$$

and

$$D^{+}={\text{max}}_{1\leq i\leq n}\left\{\frac{i}{n}-\hat{F}\left(y_{i}\right)\right\}.$$

The CvM test statistic is

$$W^{2}=\sum_{i=1}^{n}{\left[\hat{F}\left(y_{i}\right)-\frac{\left(2i-1\right)}{2n}\right]}^{2}+\frac{1}{12}n,$$ (16)

and the AD test statistic is

$$A^{2}=-n-\frac{1}{n}\overset{n}{\underset{i=1}{\sum }}\left(2i-1\right)\left\{log\left[\hat{F}\left(y_{i}\right)\right]+log\left[1-\hat{F}\left(y_{n+1-i}\right)\right]\right\}.$$ (17)

Note that the estimate $\hat{F}$ of F is obtained by substituting the estimated parameter $\hat{w}$ for *w* in (6).

### 5.2. Critical values

When all three parameters are unknown, the problem is reduced to a testing whether the *y* values have distribution (5). Because the distribution of *y* is only related to *w*, we can obtain the critical values of three GoF tests under a given *w* in practice [11]. Critical values for the GoF test statistics are obtained similarly as those for the PPC test. For *w* = 0.057, the algorithm to obtain the 5% critical values for the GoF test statistics is as follows:

(i) Set *w* = 0.057, generate *n* random numbers from (7), and order the sample values to obtain *y*_1_ ≤ *y*_2_ ≤ ⋯ ≤ *y*_*n*_.
(ii) Obtain the estimated parameters $\hat{w}$ for *w* by maximum likelihood.
(iii) Calculate the GoF test statistics *D*, *W*^2^, and *A*^2^ using (15)–(17), respectively.
(iv) Repeat steps (i)–(iii) 20 000 times. Then, obtain the 5% sample quantiles of *D*, *W*^2^, and *A*^2^ as the 5% critical values.

Table 3 presents the critical values of the distribution of *D* for selected sample sizes at five significance levels when *w* equals 0.057. For example, at a 5% significance level, the critical value of *D* for *n* = 10 is 0.408884; this means that in 5% of random samples of size 10, the maximum absolute deviation between the sample and population cumulative distributions will be at least 0.408884. Tables 4 and 5 present the respective critical values of *W*^2^ and *A*^2^.

**Table 3:** Critical values of Kolmogorov–Smirnov test statistic *D*.

*n*	0.20	0.15	0.10	0.05	0.01
**5**	0.446880	0.473885	0.509393	0.566371	0.672093
**10**	0.323630	0.343660	0.369847	0.408884	0.485664
**15**	0.267126	0.283803	0.306051	0.339473	0.403396
**20**	0.231045	0.245980	0.264881	0.294413	0.350114
**25**	0.207668	0.220027	0.237144	0.263833	0.315343
**30**	0.191299	0.202867	0.218438	0.243073	0.290677
**35**	0.175117	0.185833	0.200507	0.223375	0.266732
**40**	0.165582	0.175921	0.189560	0.210105	0.250014
**45**	0.156048	0.165915	0.178628	0.198491	0.238380
**50**	0.149112	0.158542	0.170159	0.189142	0.227326
**55**	0.140810	0.149681	0.161110	0.178707	0.216267
**60**	0.136262	0.144449	0.155538	0.172211	0.206716
**65**	0.130565	0.138945	0.149420	0.166197	0.198775
**70**	0.126203	0.134143	0.144402	0.159965	0.193367
**75**	0.121980	0.129378	0.139224	0.154886	0.187107
**80**	0.117798	0.124973	0.134668	0.148995	0.179432
**85**	0.114445	0.121868	0.130726	0.145745	0.175448
**90**	0.111515	0.118039	0.127052	0.141222	0.169329
**100**	0.105823	0.112233	0.121002	0.134505	0.159930
**150**	0.086871	0.091964	0.099259	0.110543	0.133884
**200**	0.075353	0.079988	0.085856	0.095249	0.113816
**250**	0.067065	0.071249	0.076621	0.084715	0.101696
**300**	0.061162	0.065032	0.070101	0.078012	0.094061
**350**	0.057030	0.060426	0.064677	0.071959	0.086416
**400**	0.053144	0.056409	0.060820	0.067380	0.080269
**450**	0.050350	0.053380	0.057352	0.063812	0.076789
**500**	0.047571	0.050407	0.054202	0.060744	0.072821
**600**	0.043396	0.046120	0.049547	0.055057	0.065840
**700**	0.040375	0.042854	0.046188	0.051181	0.062073
**800**	0.037704	0.040059	0.043098	0.048173	0.058031
**900**	0.035729	0.037811	0.040733	0.045370	0.054036
**1000**	0.033735	0.035836	0.038548	0.042769	0.051306

**Table 4:** Critical values of Cramér–von Mises test statistic *W*^2^.

*n*	0.20	0.15	0.10	0.05	0.01
**5**	0.242335	0.280943	0.340421	0.449563	0.683978
**10**	0.244610	0.288169	0.346830	0.459140	0.723887
**15**	0.244047	0.290599	0.353364	0.464362	0.734832
**20**	0.239459	0.280191	0.343665	0.455073	0.721312
**25**	0.241777	0.282304	0.344065	0.454116	0.744248
**30**	0.242533	0.287304	0.349097	0.461176	0.734066
**35**	0.238571	0.279211	0.341457	0.453184	0.721340
**40**	0.242236	0.284130	0.347295	0.459823	0.742361
**45**	0.241033	0.281383	0.344585	0.454730	0.751267
**50**	0.243571	0.283901	0.344627	0.458690	0.753552
**55**	0.239484	0.281403	0.344137	0.455080	0.744110
**60**	0.243420	0.286339	0.349246	0.462410	0.717814
**65**	0.241504	0.284029	0.347463	0.464705	0.734423
**70**	0.245412	0.286896	0.348701	0.462172	0.759487
**75**	0.243716	0.284682	0.347253	0.463393	0.747111
**80**	0.239299	0.283146	0.347169	0.462007	0.739443
**85**	0.242573	0.283551	0.347537	0.458651	0.742567
**90**	0.243841	0.284719	0.345539	0.459457	0.764940
**100**	0.241622	0.286697	0.352003	0.467149	0.736139
**150**	0.242416	0.286902	0.354309	0.466888	0.777429
**200**	0.243170	0.285139	0.347693	0.458361	0.742243
**250**	0.239284	0.281556	0.343206	0.461385	0.721397
**300**	0.238194	0.280306	0.345560	0.457530	0.754071
**350**	0.238976	0.280971	0.345150	0.459912	0.766382
**400**	0.239944	0.282440	0.344317	0.457545	0.736096
**450**	0.243716	0.286146	0.349780	0.461976	0.744214
**500**	0.240597	0.285261	0.348788	0.463800	0.753251
**600**	0.238319	0.285524	0.348505	0.460363	0.728151
**700**	0.241458	0.286305	0.350914	0.469165	0.759007
**800**	0.241763	0.284923	0.352221	0.467426	0.768439
**900**	0.243253	0.285066	0.349809	0.466835	0.751690
**1000**	0.242073	0.284999	0.347739	0.461170	0.751950

**Table 5:** Critical values of Anderson–Darling test statistic *A*^2^.

*n*	0.20	0.15	0.10	0.05	0.01
**5**	1.402368	1.617315	1.948235	2.540807	3.945985
**10**	1.432449	1.638524	1.952050	2.534949	3.975670
**15**	1.423500	1.655345	1.989913	2.542914	3.968316
**20**	1.397534	1.604287	1.944155	2.487906	3.883521
**25**	1.411099	1.614757	1.938555	2.498803	3.940799
**30**	1.414994	1.633178	1.949956	2.508170	3.902165
**35**	1.391693	1.596892	1.893436	2.465529	3.867444
**40**	1.408786	1.620008	1.939128	2.496215	3.870322
**45**	1.409241	1.616620	1.938735	2.485229	3.968667
**50**	1.407365	1.616091	1.924136	2.497151	3.955717
**55**	1.396348	1.606478	1.920550	2.482479	3.976492
**60**	1.418677	1.639344	1.947566	2.505852	3.783002
**65**	1.415882	1.622051	1.939137	2.504478	3.913950
**70**	1.422956	1.638156	1.940031	2.500971	3.999977
**75**	1.421766	1.621398	1.955952	2.504901	3.839541
**80**	1.400317	1.617403	1.947304	2.529683	3.918236
**85**	1.404970	1.628421	1.931324	2.478782	3.923780
**90**	1.413251	1.622044	1.930072	2.475071	3.984068
**100**	1.407514	1.628714	1.948187	2.522163	3.859779
**150**	1.414392	1.629372	1.967651	2.544987	4.031543
**200**	1.421308	1.624960	1.924006	2.471705	3.956823
**250**	1.395458	1.604342	1.916751	2.483355	3.765097
**300**	1.398433	1.613121	1.929136	2.467790	3.925358
**350**	1.397161	1.614259	1.912871	2.498783	3.985258
**400**	1.399611	1.607549	1.919304	2.494828	3.891362
**450**	1.411394	1.630574	1.939966	2.502965	3.919856
**500**	1.411623	1.632993	1.944294	2.522671	3.927478
**600**	1.399296	1.617759	1.936720	2.483390	3.831136
**700**	1.402176	1.628733	1.951434	2.503491	3.965035
**800**	1.407660	1.623932	1.947692	2.500206	3.973417
**900**	1.405134	1.627551	1.946872	2.511161	3.930996
**1000**	1.407513	1.623074	1.936012	2.492179	3.938123

For the GoF tests, the null hypothesis is that the sample comes from a PFLC distribution, and the alternative hypothesis is that it does not. We can determine whether the PFLC distribution can be used to fit one dataset by GoF tests at 5% significance level as follows:

(i) Order the sample values to obtain *x*_1_ ≤ *x*_2_ ≤ ⋯ ≤ *x*_*n*_.
(ii) Obtain the estimated parameters $\left(\hat{\alpha },\hat{\theta },\hat{\sigma }\right)$ for (*α*, *θ*, *σ*) by maximum likelihood, and calculate $\hat{w}={\left\{1+\sqrt{2\pi }\hat{\alpha }\hat{\sigma }\Phi \left(\hat{\alpha }\hat{\sigma }\right)exp\left[0.5{\left(\hat{\alpha }\hat{\sigma }\right)}^{2}\right]\right\}}^{-1}$.
(iii) Calculate $y_{i}=\hat{w}{\left(x_{i}/\hat{\theta }\right)}^{\hat{\alpha }},i=1,2,\cdots ,n$.
(iv) Calculate GoF test statistics *D*, *W*^2^, and *A*^2^ using (15)–(17), respectively.
(v) Reject *H*_0_ (the sample is from a PFLC distribution) at 5% significance level if the statistic exceeds the 5% critical values.

### 5.3. Power comparison

The power of a test is the probability that it will reject the null hypothesis if the alternative hypothesis is true (hence, power is the complement of the probability of a Type II error). Therefore, the power depends on the alternative distribution. A Monte Carlo study was performed to evaluate the power of the three GoF tests using 100 000 samples of different sizes from four alternative distributions. For these tests, the null hypothesis was that generated observations were drawn from a PFLC distribution (4,5,0.42). Note that the mixing weight *w* is 0.057 for PFLC(4,5,0.42), and the 5% critical values are from Tables 3–5. Simulations were carried out in MATLAB and all the codes used can be found from supporting information.

Four alternative distributions were considered: Gamma (3.5, 2.7), *χ*^2^ (10), LN(2.3, 0.5), and Weibull(10, 2), whose PDFs are as follows:

$$\text{Gamma}\left(a,b\right)=\frac{1}{b^{a}\Gamma \left(a\right)}x^{a-1}e^{-\frac{x}{b}},$$

$$\chi^{2}\left(\upsilon \right)=\frac{1}{2^{\upsilon /2}\Gamma \left(\upsilon /2\right)}x^{\left(\upsilon -2\right)/2}e^{-\frac{x}{2}},$$

$$\text{LN}\left(\mu ,\sigma \right)=\frac{1}{x\sigma \sqrt{2\pi }}exp\left\{-\frac{{\left(lnx-\mu \right)}^{2}}{2\sigma^{2}}\right\},$$

$$\text{Weibull}\left(a,b\right)=\frac{b}{a}{\left(\frac{x}{a}\right)}^{b-1}e^{-{\left(x/a\right)}^{b}}.$$

**Fig 3:**
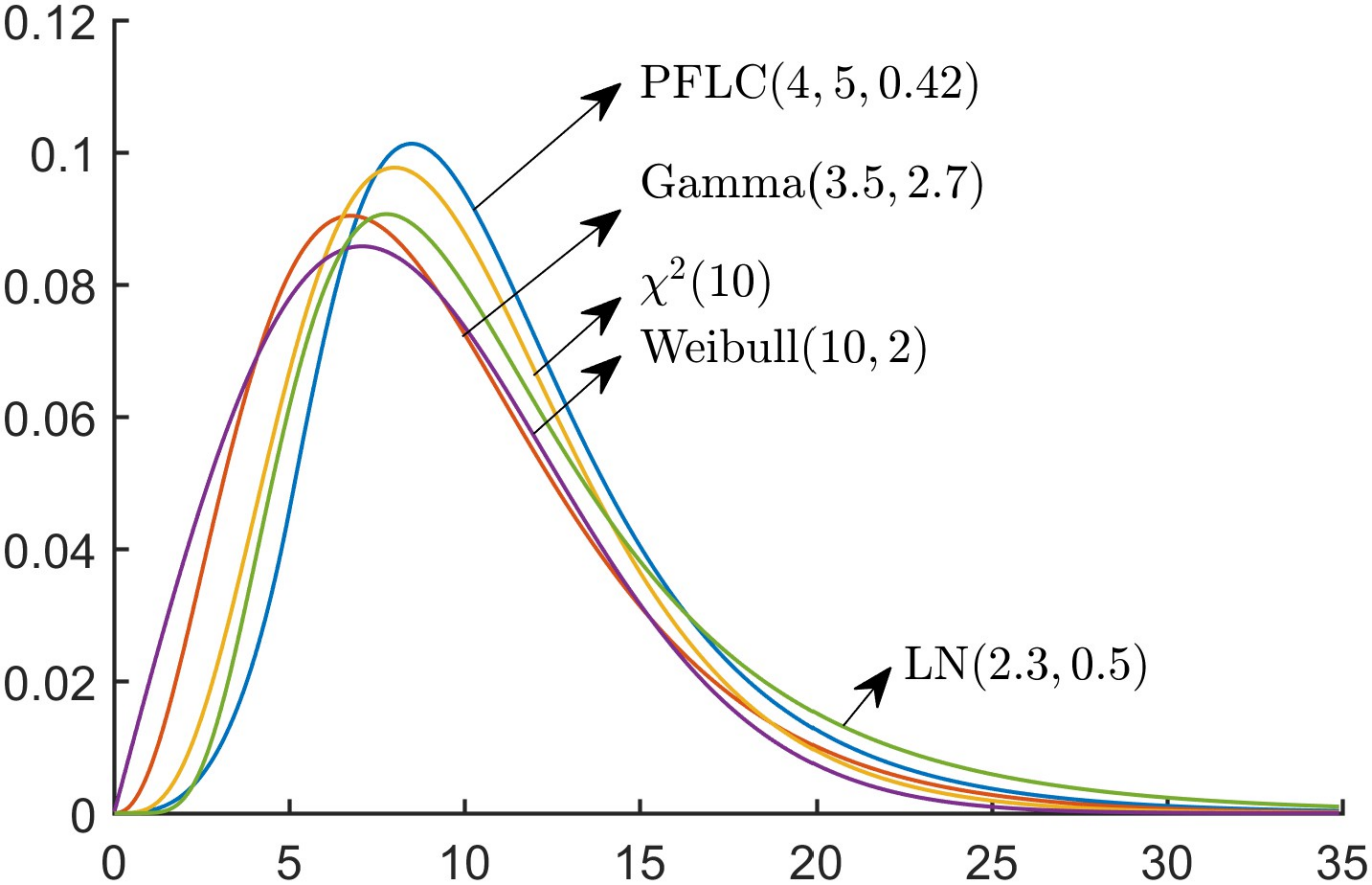
Plots of PFLC (4,5,0.42) and four alternative distributions.

The plots of five distributions are shown in Fig 3, from which it can be seen that PFLC(4,5,0.42) has the largest mode. For the left side of the mode of PFLC(4,5,0.42), all of the distributions have a slight discrepancy, while for the right side, it is difficult to distinguish them from the PDFs. Thus, these five distributions exhibit similar overall shapes. We note that if the shapes of these five distributions differ significantly, then the results will be highly unreliable.

Table 6 summarizes the simulated powers of a PPC test for four selected distributions at the 5% significance level, which can be seen to increase with the sample sizes for the same alternative distribution.

**Table 6:** Observed power of PPC test at 5% significance level when the true population is FPLC (4,5,0.42).

*n*	Gamma(3.5,2.7)	χ^2^(10)	LN(2.3,0.5)	Weibull(10,2)
**10**	0.0547	0.0497	0.0298	0.1192
**20**	0.0661	0.0539	0.0310	0.1936
**40**	0.0835	0.0618	0.0333	0.3429
**60**	0.1156	0.0715	0.0342	0.5135
**80**	0.1229	0.0805	0.0357	0.6148
**100**	0.1408	0.0828	0.0424	0.7145
**150**	0.1971	0.1078	0.0431	0.8843
**200**	0.2656	0.1429	0.0634	0.9576
**400**	0.5288	0.2828	0.1642	0.9996
**600**	0.7513	0.4583	0.2908	1.0000
**800**	0.8697	0.5969	0.4059	1.0000
**1000**	0.9457	0.7396	0.6408	1.0000

Table 7 summarizes the simulated power for four selected distributions at the 5% significance level. From Table 7, the following can be seen:

(i) The powers of the tests increase with the sample sizes.
(ii) The AD test outperforms the KS and CvM tests across different sizes and alternative distributions.
(iii) The KS test is the weakest test, and it requires a much larger sample to achieve comparable power to the other two tests.
(iv) The powers of the tests are the smallest when the alternative distribution is LN(2.3, 0.5), which is the most similar to PFLC(4,5,0.42), as can be seen from Fig 3.

**Table 7:** Observed power of GoF tests at 5% significance level when the true population is FPLC (4,5,0.42).

*n*	Test	Gamma(3.5,2.7)	χ^2^(10)	LN(2.3,0.5)	Weibull(10,2)
**10**	***D***	0.2008	0.0864	0.0679	0.2500
	***W*** ^**2**^	0.2151	0.0896	0.0647	0.2779
	***A*** ^**2**^	0.3103	0.1088	0.0912	0.4211
**20**	***D***	0.3306	0.1110	0.0717	0.4094
	***W*** ^**2**^	0.3655	0.1334	0.0769	0.4650
	***A*** ^**2**^	0.5005	0.1618	0.1099	0.6461
**40**	***D***	0.5599	0.1815	0.0927	0.7031
	***W*** ^**2**^	0.5954	0.2175	0.0878	0.7508
	***A*** ^**2**^	0.7469	0.2671	0.1406	0.8920
**60**	***D***	0.7476	0.2416	0.1017	0.8654
	***W*** ^**2**^	0.7738	0.2915	0.0962	0.8929
	***A*** ^**2**^	0.8901	0.3554	0.1662	0.9683
**80**	***D***	0.8648	0.3171	0.1169	0.9480
	***W*** ^**2**^	0.8743	0.3748	0.1066	0.9593
	***A*** ^**2**^	0.9504	0.4510	0.1888	0.9931
**100**	***D***	0.9254	0.3624	0.1325	0.9800
	***W*** ^**2**^	0.9350	0.4337	0.1220	0.9847
	***A*** ^**2**^	0.9819	0.5247	0.2286	0.9983
**150**	***D***	0.9884	0.5186	0.1639	0.9995
	***W*** ^**2**^	0.9891	0.6110	0.1564	0.9995
	***A*** ^**2**^	0.9987	0.7079	0.3163	1.0000
**200**	***D***	0.9988	0.6469	0.2136	1.0000
	***W*** ^**2**^	0.9989	0.7371	0.2142	1.0000
	***A*** ^**2**^	0.9998	0.8339	0.4375	1.0000
**400**	***D***	1.0000	0.9230	0.4229	1.0000
	***W*** ^**2**^	1.0000	0.9602	0.4632	1.0000
	***A*** ^**2**^	1.0000	0.9870	0.7685	1.0000
**600**	***D***	1.0000	0.9868	0.6349	1.0000
	***W*** ^**2**^	1.0000	0.9957	0.7116	1.0000
	***A*** ^**2**^	1.0000	0.9997	0.9382	1.0000
**800**	***D***	1.0000	0.9983	0.7974	1.0000
	***W*** ^**2**^	1.0000	0.9993	0.8733	1.0000
	***A*** ^**2**^	1.0000	0.9999	0.9863	1.0000
**1000**	***D***	1.0000	0.9998	0.9058	1.0000
	***W*** ^**2**^	1.0000	1.0000	0.9537	1.0000
	***A*** ^**2**^	1.0000	1.0000	0.9976	1.0000

Hence, we recommend the AD test, followed by the CvM and KS tests.

## 6. Real data analysis

We consider an example to illustrate the methods discussed in Sections 3–5. The real dataset (BREAST) represents the survival times of 121 patients with breast cancer, as obtained from a large hospital from 1929 to 1938 [6], as follows:

0.3, 0.3, 4.0, 5.0, 5.6, 6.2, 6.3, 6.6, 6.8, 7.4, 7.5, 8.4, 8.4, 10.3, 11.0, 11.8,12.2, 12.3, 13.5, 14.4, 14.4, 14.8, 15.5, 15.7, 16.2, 16.3, 16.5, 16.8, 17.2, 17.3, 17.5, 17.9, 19.8, 20.4,20.9, 21.0, 21.0, 21.1, 23.0, 23.4, 23.6, 24.0, 24.0, 27.9, 28.2, 29.1, 30.0, 31.0, 31.0, 32.0, 35.0, 35.0,37.0, 37.0, 37.0, 38.0, 38.0, 38.0, 39.0, 39.0, 40.0, 40.0, 40.0, 41.0, 41.0, 41.0, 42.0, 43.0, 43.0, 43.0,44.0, 45.0, 45.0, 46.0, 46.0, 47.0, 48.0, 49.0, 51.0, 51.0, 51.0, 52.0, 54.0, 55.0, 56.0, 57.0, 58.0, 59.0, 60.0, 60.0, 60.0, 61.0, 62.0, 65.0, 65.0, 67.0, 67.0, 68.0, 69.0, 78.0, 80.0, 83.0, 88.0, 89.0, 90.0, 93.0,96.0, 103.0, 105.0, 109.0, 109.0, 111.0, 115.0, 117.0, 125.0, 126.0, 127.0, 129.0, 129.0, 139.0, 154.0.

Table 8 shows the summary statistics for the BREAST data. The mean is 46.3289, and the median is 40.00. Notice that the mean is greater than the median, indicating that the distribution of data is likely skewed to the right.

**Table 8:** Summary statistics for BREAST data.

Statistic	Value
Total Observations	121
Mean	46.3289
Standard Deviation	35.2770
Minimum	0.30
Maximum	154
First Quartile	17.45
Median	40.00
Third Quartile	60.25
Skewness	1.0432
Kurtosis	3.4021

**Fig 4:**
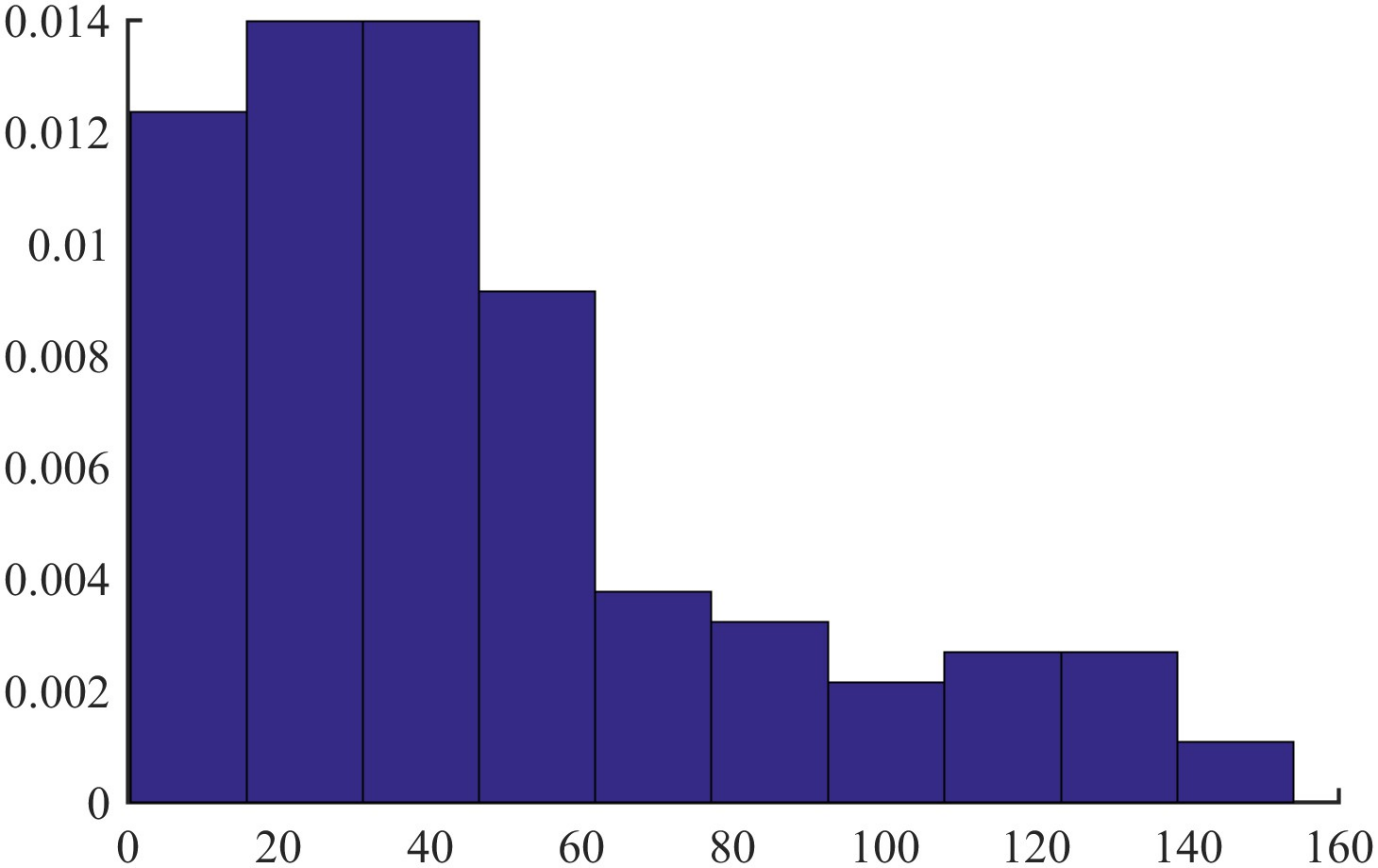
Histogram of BREAST data.

Fig 4 shows a histogram of these data, whose left side seems “chopped off” compared to the right side, which we describe as being skewed to the right.

**Fig 5:**
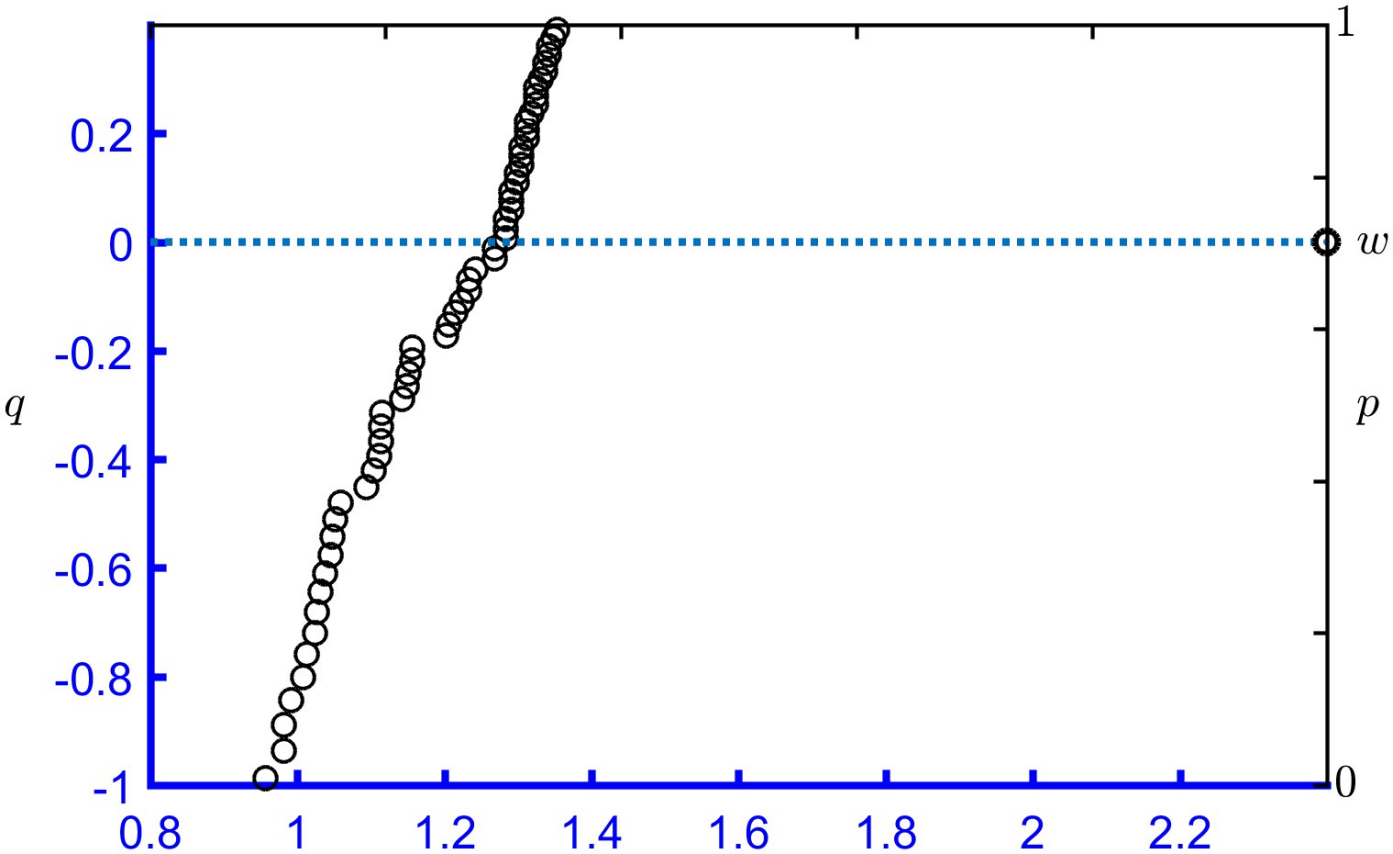
Probability plot of BREAST data.

In this study, the PFLC maximum likelihood estimates are $\hat{\alpha }=1.05,\hat{\theta }=34.10$, and $\hat{\sigma }=0.5749$. Fig 5 presents the probability plot of the BREAST data, whose points approximate a straight line, which means that the PFLC distribution can provide a good fit to these data.

**Fig 6:**
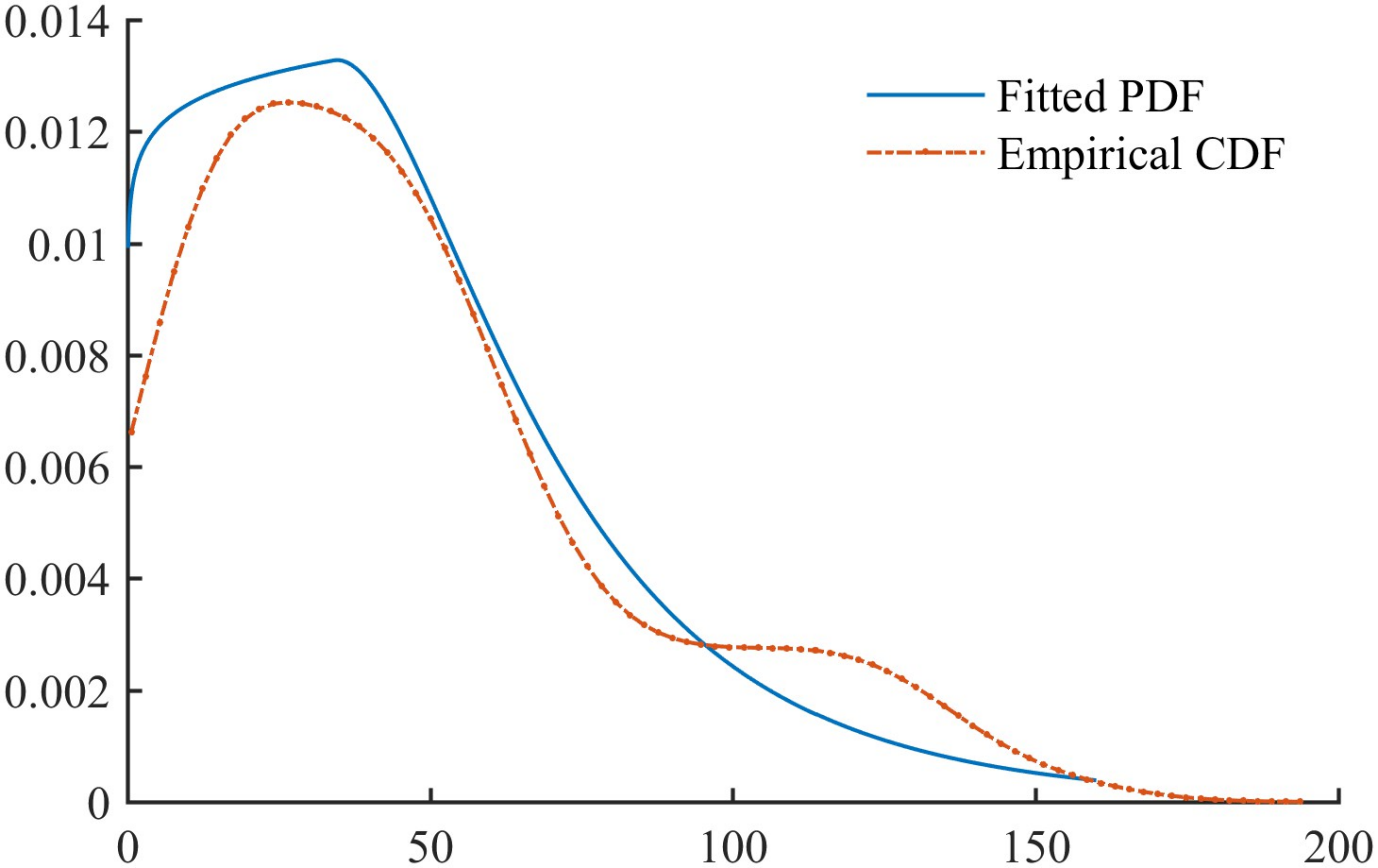
Empirical and fitted PDFs for BREAST data.

**Fig 7:**
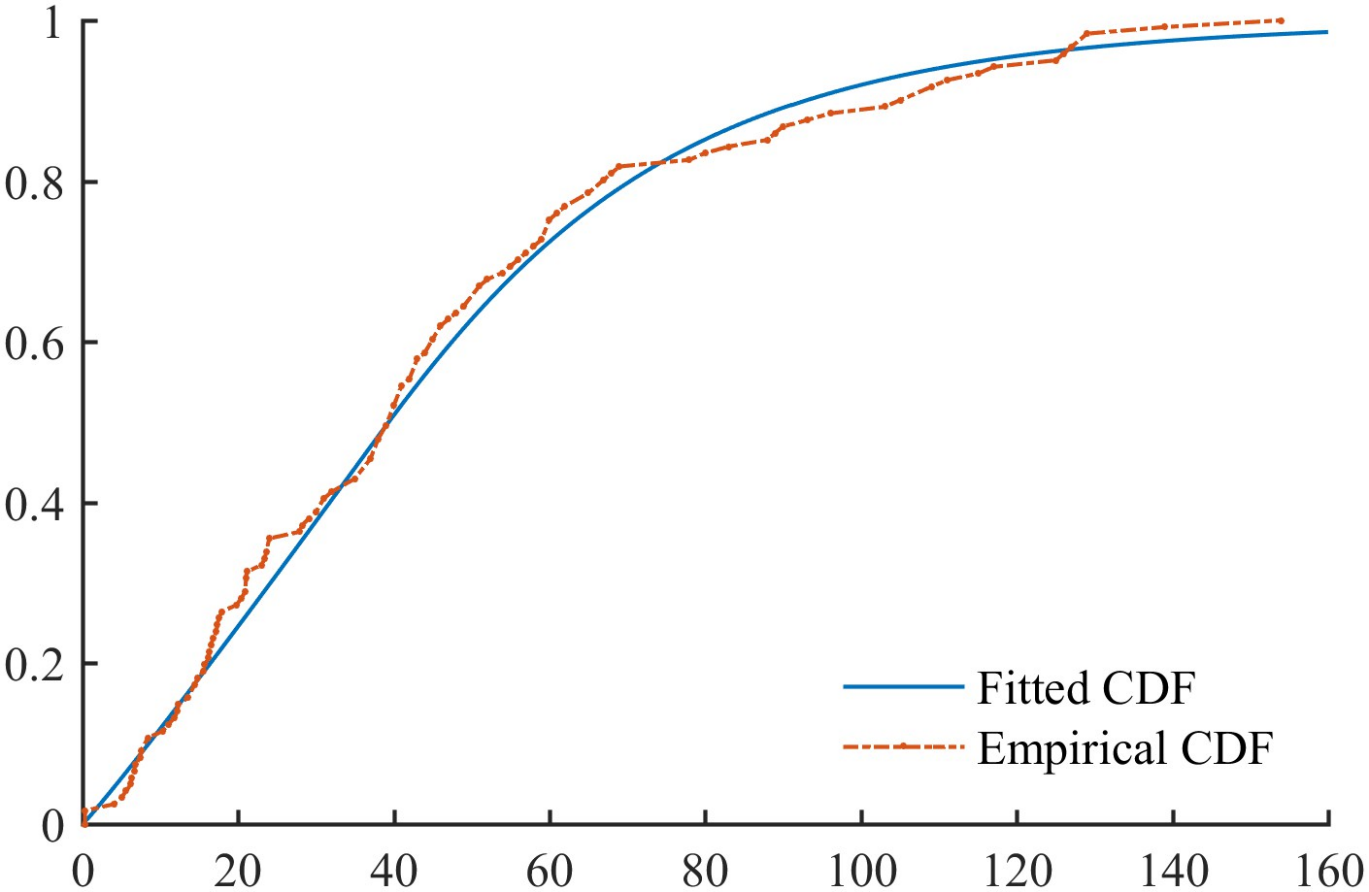
Empirical and fitted CDFs for BREAST data.

Fig 6 compares the fitted and empirical PDF of the BREAST data, and Fig 7 compares the fitted and empirical CDF of the BREAST data. It can be seen that the PFLC CDF exhibits a good match to the empirical CDF, but there is a slight discrepancy between the empirical and PFLC PDFs.

Table 9 reports the test statistics and p–values (in parentheses) for the PPC and three GoF tests. It indicates that the PFLC distribution provides a reasonable fit to the BREAST data.

**Table 9:** Tests and associated p–values for BREAST data.

Test	PPC	AD	CvM	KS
Statistic (p-value)	0.9835 (0.1271)	0.4832 (0.6525)	0.0592 (0.5706)	0.0573 (0.8225)

## 7. Conclusions

Goodness–of–fit testing is a key procedure for selecting the statistical distribution that best fits observed data. We performed tests of fit for the PFLC distribution, including the probability plot, probability plot correlation coefficient, KS test, CvM test, and AD test. Among these methods, the probability plot is a graphical method, while the others are statistical methods. We described the probability plot and considered the procedures, algorithms, and critical values for the other methods. We found that the AD test outperformed KS and CvM tests based on power comparisons. Moreover, this new PFLC distribution was first successfully used to model the survival times of breast cancer patients. The tests developed in this paper revealed that PFLC fits the data well. The work in this paper can be extended in some ways, such as to: (i) calculate the lifetime statistics, such as the hazard rate and the mean residual life based on the PFLC distribution; (ii) develop other GoF tests methods, such as Chi-squared tests and likelihood ratios; (iii) compare the performance of the PFLC distribution with other competitive alternatives; and (iv) investigate its applications in other disciplines.

## Supporting information

S1 File Codes used in MATLAB. (ZIP)

## References

[1] Kleiber C, Kotz S. Statistical Size Distributions in Economics and Actuarial Sciences. New York: John Wiley; 2003.

[2] Wang C. Parameter estimation for power function lognormal composite distribution. Communications in Statistics—Theory and Methods. 2023; 52(9):2966–2982. doi: 10.1080/03610926.2021.1965622

[3] Wang C. Inequality measures based on log power function lognormal composite distribution. Applied Economics Letters. 2022; 1–6. doi: 10.1080/13504851.2022.2130862

[4] Sarhan AM, Ahmad AA, Alasbahi IA. Exponentiated generalized linear exponential distribution. Applied Mathematical Modelling. 2013; 37(5):2838–49. doi: 10.1016/j.apm.2012.06.019

[5] Nofal ZM, Afify AZ, Yousof HM, Gauss MC. The generalized transmuted-G family of distributions[J]. Communications in Statistics-Theory and Methods. 2017; 46(8): 4119–4136. doi: 10.1080/03610926.2015.1078478

[6] Awodutire PO, Balogun OS, Olapade AK, Nduka EC. The modified beta transmuted family of distributions with applications using the exponential distribution. PloS ONE. 2021; 16(11): e0258512. doi: 10.1371/journal.pone.0258512 34793462 PMC8601563

[7] Fayomi A, Khan S, Tahir MH, Algarni A, Jamal F, Abu-Shanab R. A new extended gumbel distribution: Properties and application. PLoS ONE. 2022; 17(5): e0267142. doi: 10.1371/journal.pone.0267142 35622822 PMC9140309

[8] Ozkan E, Golbasi Simsek G. Generalized Marshall-Olkin exponentiated exponential distribution: Properties and applications[J]. PloS ONE. 2023; 18(1): e0280349. doi: 10.1371/journal.pone.0280349 36652462 PMC9847959

[9] Kimball BF. On the choice of plotting positions on probability paper. Journal of the American Statistical Association.1960; 55(291):546–560. doi: 10.1080/01621459.1960.10482081

[10] Filliben JJ. The Probability Plot Correlation Coefficient Test for Normality. Technometrics. 1975; 17(1):111–117. doi: 10.1080/00401706.1975.10489279

[11] D’Agostino R, Stephens MA. Goodness-of-Fit Techniques. New York: Taylor & Francis; 1986.

